# Scalp necrotizing fasciitis after resection of angiolymphoid hyperplasia with eosinophilia misdiagnosed as pyoderma gangrenosum

**DOI:** 10.1016/j.jdcr.2025.10.013

**Published:** 2025-10-16

**Authors:** Yuting Zhou, Liwei Ran, Long Zhang, Zhenzhen Ye, Xin Yang, Ye Liu, Chen Wang, Yingying Yan, Weiwei Li, Jinzhu Guo, Dirk M. Elston, Wen-Hui Wang

**Affiliations:** a Department of Dermatology, Peking University Third Hospital, Haidian District, Beijing; b Department of Dermatology, Beijing Chao-Yang Hospital, Capital Medical University, Chaoyang District, Beijing; c Wound Healing Center, Peking University Third Hospital, Haidian District, Beijing; d Department of Plastic Surgery, Peking University Third Hospital, Haidian District, Beijing; e Department of Endocrinology, Peking University Third Hospital, Haidian District, Beijing; f Department of Nutrition, Peking University Third Hospital, Haidian District, Beijing; g Department of Pharmacy, Peking University Third Hospital, Haidian District, Beijing; h Department of Dermatology and Dermatologic Surgery Medical University of SC, Charleston, South Carolina

**Keywords:** angiolymphoid hyperplasia with eosinophilia (ALHE), necrotizing fasciitis, pyoderma gangrenosum, recurrent scalp ulcer, refractory wound management

## Introduction

Angiolymphoid hyperplasia with eosinophilia (ALHE) is a rare, benign vascular proliferative disorder typically characterized by solitary or multiple red papules or nodules, most commonly occurring in the head and neck region.^1^ Although its precise etiology remains unclear, proposed triggers include prior trauma, infections, reactive hyperplasia, and hyperestrogenemia.^2^ Ulcerative or destructive forms are uncommon and may mimic other inflammatory or infectious dermatoses. Here, we present a case of scalp necrotizing fasciitis occurring after excision of ALHE that was initially misdiagnosed as pyoderma gangrenosum (PG), highlighting diagnostic pitfalls and the need for careful clinicopathologic correlation in ulcerative presentations.

## Case report

**Fig 1:**
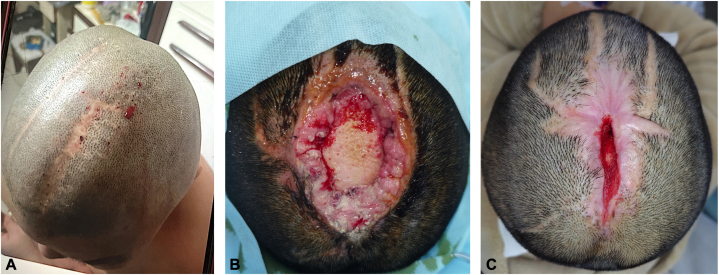
Clinical course and progression of recalcitrant scalp ulcer. **A,** 3 Months prior to admission, before the third excision: spontaneous punctate bleeding with significant pain and swelling sensation, similar appearance before the previous 2 excisions. **B,** On admission: large ulcer (8 × 6 cm) with exposed bone, adherent necrotic debris, purulent exudate, and steep inflammatory margins. **C,** Healing phase after antimicrobial therapy, wound hygiene, and platelet-rich plasma applications.

A 34-year-old woman presented to the dermatology department with recurrent scalp hemorrhage and ulceration for 3 years, worsening over the past 3 months. Three years ago, the patient felt a significant swelling sensation and pain on the vertex scalp, and developed spontaneous punctate bleeding and hemorrhagic ulcerations. After tolerating for 8 months, she underwent the initial surgical excision and achieved 9 months’ remission. However, the condition recurred later, and she tolerated it for 2 weeks before undergoing a second excision and achieving 1-year remission. Three months prior to the current admission, the condition recurred again, and she underwent a third excision (Fig 1, *A*), after which the wound failed to heal. Debridement and flap grafting were attempted, but the ulcer area continued to enlarge with severe pain. PG was suspected, and she received oral tofacitinib (5 mg twice daily for 10 days) followed by intravenous methylprednisolone (80 mg once daily for 17 days). But her symptoms worsened, leading to admission to our hospital. No significant weight changes occurred during the disease course.

**Fig 2:**
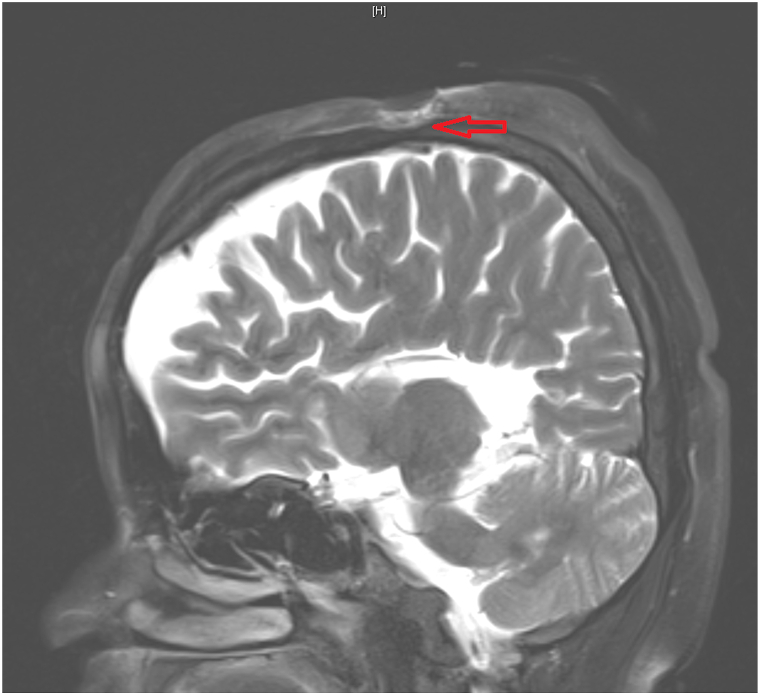
Cranial magnetic resonance imaging showing a deep vertex scalp ulcer (red arrow) extending to and involving the calvarial outer table.

**Fig 3:**
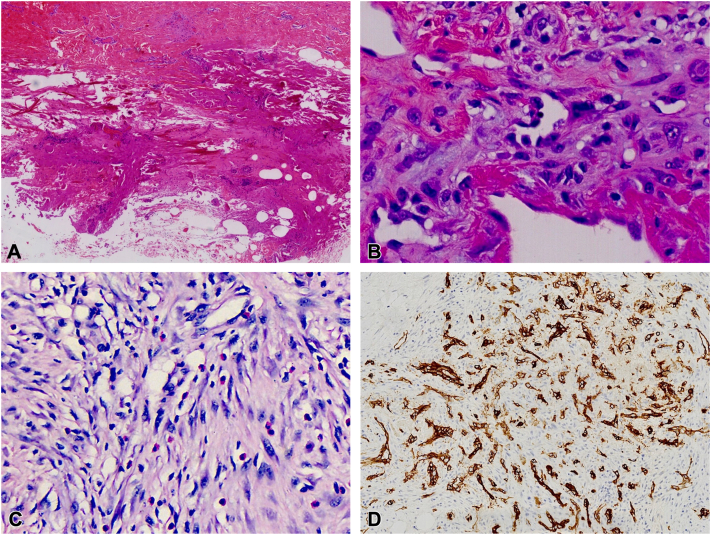
Histopathological features of excised scalp lesions. **A,** Base of the third excision specimen showing necrotic debris and acute inflammation, suggestive of necrotizing fasciitis. **B** and **C,** Lobular proliferation of small vessels with endothelial cells protruding into the lumens and scattered intracytoplasmic vacuoles; fibrous stroma with sparse lymphocytic infiltration and eosinophils. **D,** Immunohistochemistry: endothelial cells stained positive for CD34 in proliferating vessels (×20). (**A-C** Hematoxylin-eosin stain; original magnifications: **A,** ×4; **B,** ×40; **C,** ×40.) (**D,** original magnification: **D,** ×20.)

On presentation to our hospital, we noted an ulcer measuring approximately 8 × 6 cm, extending down to the bone. Cranial magnetic resonance imaging demonstrated a deep vertex ulcer with involvement of the outer table of the calvarium (Fig 2). The ulcer base consisted predominantly of exposed bone, with minimal edematous granulation tissue present at the margins. Edges were steep, featuring irregular inflammatory hyperplasia with adherent necrotic debris and purulent exudate. Linear postoperative scars surrounded the lesion; the remainder of the skin was unremarkable (Fig 1, *B*). Peripheral eosinophilia was noticed with an absolute value: 0.64 × 10^9^/L (10.3%). Scalp exudate and tissue cultures both yielded *Proteus mirabilis* and *Morganella morganii*. Review of histopathologic specimen of the 3 excisions revealed lobular proliferation of small vessels, with the endothelial cells protruding into the lumens and scattered intracytoplasmic vacuoles. The stroma was fibrous and exhibited sparse lymphocytic infiltration and scattered eosinophils (Fig 3, *B* and *C*). The endothelial cells stained positive for CD34 (Fig 3, *D*), and smooth muscle actin staining highlighted a continuous pericytic layer surrounding the proliferating vessels. No rearrangement was detected using a pan-sarcoma next-generation sequencing panel.

Systemic glucocorticoids were tapered over 3 weeks, and the ulcer gradually decreased in size with antimicrobial therapy, sustained wound hygiene, gentle debridement, and platelet-rich plasma applications. The patient was discharged after 2 months, and the ulcer was essentially healed within 3 months. However, 1 month after the wound closure, the lesion recurred with worsened ulceration and pain, necessitating readmission. Following 2 additional months of similar wound management, the ulcer showed marked healing (Fig 1, *C*). During hospitalization, critical hypophosphatemia developed (serum phosphate 0.43 mmol/L ↓↓; serum calcium 2.19 mmol/L ↓; urinary calcium: 2.06 mmol/24h ↓; and urinary phosphate: 24.38 mmol/24h ↓). A phosphaturic mesenchymal tumor was suspected, but PET-CT revealed no additional neoplastic foci, and the lesional tissue never revealed features of this tumor. The electrolyte imbalance was attributed to nutritional deficiency and refeeding and corrected with phosphate and calcium supplementation.

## Discussion

The histologic changes in the initial, painful punctate bleeding lesion were reactive with features of granulation tissue and ALHE,^3^ which may be induced by trauma or folliculitis. ALHE is usually located in the head and neck region and is known for its tendency to recur following surgical excision, as observed in our patient.^1^ The subsequent clinical course, characterized by a worsening ulcer and histologic evidence of fibrinous necrosis at the base of the third excision specimen (Fig 3, *A*), was more suggestive of necrotizing fasciitis than PG. This interpretation is further supported by the patient's effective improvement following systemic antibiotics and local wound care, rather than immunosuppressive therapy.^4^^,^^5^ Because Kimura disease (KD) can mimic ALHE, we note that KD is a systemic inflammatory disorder typically accompanied by lymphadenopathy and renal involvement,^6^ whereas ALHE is a localized, often trauma-related vascular malformation. The clinicopathologic profile in this case was consistent with ALHE rather than KD.

Current management options for ALHE include surgical excision, corticosteroids, radiotherapy, immunosuppressive agents, and biologic therapies, with variable efficacy. Surgical resection remains the preferred approach; however, complete removal is often challenging due to the poorly defined margins of this highly vascular lesion, and recurrence rates remain high (up to 40.8%).^1^ In ulcerative variants, meticulous wound care is critical—as demonstrated in our case, where repeated excisions and flap grafting failed, yet significant healing was achieved with antimicrobial therapy, debridement, and platelet-rich plasma application.

Ulcerative ALHE with barrier disruption carries a heightened risk of secondary infection. In this patient, repeated surgical trauma and a chronic nonhealing wound likely facilitated bacterial invasion into the deep fascia, resulting in histologically confirmed necrotizing fasciitis. These findings underscore the importance of infection control and sustained wound hygiene in managing ulcerative presentations of ALHE.

This case highlights the diagnostic challenges posed by ulcerative variants of ALHE and underscores the importance of histopathologic evaluation in distinguishing between infectious, inflammatory, and neoplastic etiologies, especially recognizing necrotizing fasciitis from PG. It also emphasizes the need to consider reactive ALHE in the differential diagnosis of chronic, nonhealing scalp ulcers with vascular proliferation, particularly when accompanied by eosinophilic infiltration. We hope this case could offer valuable insight for clinicians in recognizing atypical presentations of ALHE and avoiding misdiagnosis between necrotizing fasciitis and PG.

## Conflicts of interest

None disclosed.
